# Ubiquitomics: An Overview and Future

**DOI:** 10.3390/biom10101453

**Published:** 2020-10-17

**Authors:** George Vere, Rachel Kealy, Benedikt M. Kessler, Adan Pinto-Fernandez

**Affiliations:** 1 Target Discovery Institute, Nuffield Department of Medicine, University of Oxford, Oxford OX3 7FZ, UK; george.vere@ndm.ox.ac.uk (G.V.); benedikt.kessler@ndm.ox.ac.uk (B.M.K.); 2 St Anne’s College, University of Oxford, Oxford OX2 6HS, UK; rachel.kealy@btinternet.com; 3 Centre for Medicines Discovery, Nuffield Department of Medicine, University of Oxford, Oxford OX3 7FZ, UK; 4 Chinese Academy of Medical Sciences Oxford Institute (CAMS), Nuffield Department of Medicine, University of Oxford, Oxford OX3 7FZ, UK

**Keywords:** ubiquitin, ubiquitome, ubiquitomics, mass spectrometry, proteomics

## Abstract

Covalent attachment of ubiquitin, a small globular polypeptide, to protein substrates is a key post-translational modification that determines the fate, function, and turnover of most cellular proteins. Ubiquitin modification exists as mono- or polyubiquitin chains involving multiple ways how ubiquitin C-termini are connected to lysine, perhaps other amino acid side chains, and N-termini of proteins, often including branching of the ubiquitin chains. Understanding this enormous complexity in protein ubiquitination, the so-called ‘ubiquitin code’, in combination with the ∼1000 enzymes involved in controlling ubiquitin recognition, conjugation, and deconjugation, calls for novel developments in analytical techniques. Here, we review different headways in the field mainly driven by mass spectrometry and chemical biology, referred to as “ubiquitomics”, aiming to understand this system’s biological diversity.

## 1. Ubiquitous and Complex

Protein ubiquitination is a post-translational modification (PTM) that involves the reversible attachment of ubiquitin to amino acid side chains, most commonly a lysine, on the target protein [1]. Ubiquitin conjugation onto substrates is carried out by a series of three enzymatic steps, with a ubiquitin-activating enzyme (E1), a ubiquitin-conjugating enzyme (E2), and a ubiquitin ligase (E3) acting in a sequential manner. The modification can be removed by deubiquitinases (DUBs). Ubiquitin was first named for its ubiquitous expression across eukaryotes [2]. This name is more apt than ever, with ubiquitin and ubiquitin-like proteins contributing to regulation of almost all aspects of cell activity [3].

Ubiquitin-mediated biological processes are complex, owing to the highly variable nature of the ubiquitin modification. Attached ubiquitin can form adducts as mono- or polyubiquitin chains, the latter on different ubiquitin lysine residues, often in combination to form branched ubiquitin chains [4]. The variety in ubiquitination signals is achieved through a large number of ubiquitin ligases and DUBs that present numerous modes of action. There are 2 E1 enzymes, ∼40 E2 enzymes, ∼600 E3 ligases [5], and just under 100 DUBs [6] in the human genome giving a large scope for potential modification by the different enzymes. Signalling networks orchestrated by ubiquitin shows that E3s and DUBs exhibit redundancy and multiplicity. In addition, E3 ligases can interact with more than one E2 [7] so there is vast potential for different E2/E3 pairs to target different substrates and assemble diverse polyubiquitin chain structures.

The combinatorial possibilities of ubiquitin modification raise questions about their biological significance, but also challenges the limits of present analytical techniques. This review charts out recent progress that has been made in the field of ubiquitomics, and how these advances could help unravelling modes of ubiquitin modifications and subsequent biological effects.

## 2. Mapping Ubiquitination Sites on Protein Substrates

**Figure 1:**
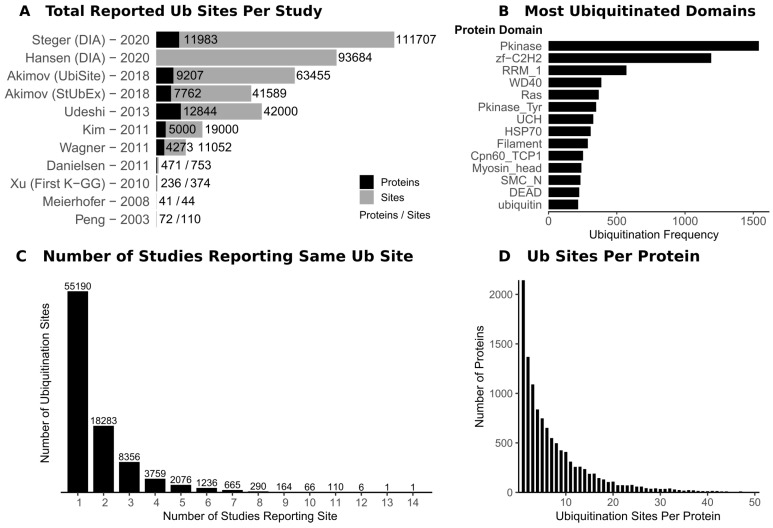
The scope of the cellular ubiquitome. (**A**) The number of ubiquitinated proteins and sites reported by key studies in the last 20 years [10,12,14,15,17,19,20,21,22,23]. (**B**–**D**) Insight from the PhosphositePlus database [24] on ubiquitinated proteins: (**B**) the most frequently ubiquitinated protein domains; (**C**) the overlap of studies reporting ubiquitination sites; and (**D**) the frequency of the number of ubiquitination sites per protein.

In addition to biochemical and antibody-based methods [8], mass spectrometry (MS)-based proteomics has enabled the rise of “ubiquitomics”, a term first coined in 2007 [9]. A ubiquitome refers to the set of proteins that are modified by ubiquitin and the associated ubiquitin chain topologies found under these conditions, although it is not possible to determine both of these aspects in the same experiment. MS-based proteomic tools for the high throughput detection of proteins which are modified with ubiquitin, referred to here as ubiquitin site profiling, have greatly increased our understanding of the ubiquitome. A difficulty that besets ubiquitin site profiling (or, indeed, the study of most PTMs) is the low stoichiometry of modification, thus an enrichment step is required prior to MS analysis. The groundwork for MS-based site specific detection of ubiquitination sites was laid in 2003, with 110 ubiquitination sites reported on 72 proteins (Figure 1A) [10]. This study relied on expression and enrichment of tagged ubiquitin in yeast followed by MS detection of the diGlycine (GG) remnant of ubiquitin, which remains conjugated to ubiquitinated proteins by an isopeptide bond to the N of a lysine side chain (K-GG) after trypsinisation.

In the decade following the study published in 2003, most ubiquitin site profiling studies relied on expression and pulldown of various forms of tagged ubiquitin, such as HA- or His-Ub [11]. While this pulldown strategy enriches ubiquitinated proteins, it suffers from skewed protein ubiquitination due to the presence of the tag, high background, and lack of definitive ubiquitination site identification. For example, Danielson et al. identified 5756 proteins following pulldown of tagged ubiquitin, but ubiquitination could only be confirmed on 471 of these proteins [12]. Other methods for investigating ubiquitin site profiling included use of biotin tagged ubiquitin [13,14], and, more recently, replacement of endogenous ubiquitin with a copy that has His-tag that remains after trypsinisation [15]. An alternative methodology, Combined Fractional Diagonal Chromatography (COFRADIC), involves monitoring chemical acetylation of lysine residues in the presence and absence of a non-specific DUB, from which >7500 ubiquitination sites were identified [16].

The biggest revolution in ubiquitin site profiling came in 2010 with the development of an antibody that that could recognise the K-GG remnant and specifically enrich for ubiquitinated material after trypsinisation [17]. The first large sale application of this antibody in 2011 enabled the identification of 19,000 sites [18]. The K-GG purification strategy has been built on in the last decade with astounding success with routine detection of ∼4000 ubiquitination sites or more in an experiment. Further optimisation has pushed this to >10,000 sites in a single run [19]. Most ubiquitin site profiling experiments rely on a commercial anti-K-GG antibody from Cell Signalling Technology.

A decade after the development of the K-GG antibody, the field is beginning to advance beyond this approach (Table 1). The K-GG antibody enrichment has its limitations. Firstly, each antibody raised against the K-GG epitope exhibits bias due to the amino acid context of the K-GG site [20]. Secondly, the K-GG antibody fails to enrich non-lysine ubiquitination modification (discussed below). Finally, the K-GG epitope is also generated following trypsinisation of ISG15- and NEDD8-modified proteins, and, while K-GG sites attributable to these modifications are low [18], these still have a confounding effect on strictly defining ubiquitination sites. One solution to these problems has been the generation of an antibody which recognises the 13-mer LysC digestion fragment of ubiquitin, referred to as the UbiSite approach [21]. LysC is typically not used alone in proteomic workflows due to its limited cleavage affinity. Additionally, detection of the 13-mer LysC fragment of ubiquitin attached to the tryptic peptide fragment derived from the substrate would be challenging due to the complex MS2 fragmentation pattern this would generate. The method therefore also uses trypsin following the pulldown to fully digest the material and generate GG-modified peptides, which are more amenable to MS detection. Across each biological triplicate, around 30,000 sites are detected and the paper reports 64,000 sites including conditions with proteasome inhibition (Figure 1A).

Methods such as stable isotope labelling of amino acids in cell culture (SILAC) [25] and tandem mass tagging (TMT) [26] have enabled multiplexing in proteomics, allowing for comparison of more conditions, decreasing sample requirements, and MS run time relative to label free quantitation approaches. Many ubiquitin site profiling experiments have used SILAC to compare 2–3 conditions such as with and without a stimulus [27] or between active and inactive ubiquitin machinery components [18]. TMT tagging permits comparison of up to 11 conditions, allowing experiments such as a detailed time course of the ubiquitination events that occur during mitophagy [28]. Performing TMT labelling on the anti-K-GG coated beads after pulldown of the GG-modified peptides enables selective labelling of the peptide N-terminus and allows TMT contaminants to be washed away, increasing the number of identified ubiquitination sites relative to in-solution TMT labelling and reducing sample requirement to sub-milligram levels. This methodology is referred to as UbiFast [29].

There are now proteomic workflows that enable the analysis of multiple “PTMomes” in single experiments through sequential pulldowns of different modifications after trypsinisation. Measurement of acetylomes and phosphoproteomes from the same sample as the ubiquitome show the interplay between PTMs and how they act together to co-ordinate cellular events [29,30,31,32]. Ubiquitome datasets increasingly include a matching proteome [21,29,32,33]. An increase in the MS quantitation value of a GG-modified peptide is more commonly explained by an increase in the abundance of its corresponding protein, so a matching proteome is necessary to tease apart differentially ubiquitinated (or otherwise modified) proteins and distinguish regulatory versus degradative ubiquitin modifications.

Two recent preprints show the application of Data-Independent Acquisition (DIA) mass spectrometry for ubiquitomics [22,23]. Although not yet peer reviewed, these studies have pushed the limits on ubiquitin site profiling, reporting ∼90,000 and ∼110,000 sites, respectively (Figure 1A). Both approaches used K-GG ubiquitin remnant enrichment combined with DIA MS to achieve deep ubiquitome coverage. Due to recent advances in software development such as DIA-NN [34], DIA MS allows improved quantitation of low abundance peptides and overcomes the stochastic nature of top-N fragmentation in Data-Dependent Acquisition (DDA) [35], which suffers from dynamic range limitations. DIA has been successfully applied to other PTMs, such as phosphorylation [36], and it seems likely that DIA MS will become common in proteomic workflows. However, DIA has limitations and the fact that there is little overlap (∼50%) between DIA and DDA ubiquitomes is concerning and shows that more work must be done to fully embrace it. There are other alternatives to DDA such as BoxCar [37] which increases the dynamic range of MS1 quantitation, but this method has seen limited use in the MS field.

## 3. Lessons from Ubiquitin Site Profiling

Ubiquitomics studies have generated a large amount of data about ubiquitination sites. While many of these papers serve to present methodological landmarks, there is insight that can be gained about the global functioning of the ubiquitin system from these datasets. There are several databases (Table 2) which have pooled information about ubiquitination sites, amongst other PTMs. This information can serve as a starting point for investigating ubiquitination of a particular protein. The PhosphositePlus database provides general information about ubiquitination, with Figure 1B highlighting the most frequently ubiquitinated domains and Figure 1D showing that substrates are generally only ubiquitinated a few times on average. However, there are outliers, with some proteins being extensively ubiquitinated, although the biological significance of this is not yet clear [21].

The interplay between ubiquitination and other PTMs has been investigated with this global data. A 2012 study exploring coevolution of PTM sites within proteins showed that ubiquitination sites frequently coevolve with phosphorylation and acetylation sites [39]. For example, phosphorylation of protein such as cyclins during the cell cycle recruits cullin-RING E3 ligases, leading to cyclin degradation by the proteasome [40]. However, this analysis only employed ∼600 ubiquitination sites, so new investigations with deeper ubiquitome data may show new trends. In the Protein Lysine Modification Database from 2017 there are 24,487 co-occurrences of acetylation and ubiquitination on the same residue [41]. Studies that profile both acetylation and ubiquitin sites have found a negative correlation between the two modifications on proteins [29]. This suggests that acetylation of a lysine residue could physically block ubiquitination at that site, this being particularly relevant in the case of epigenetic regulations [42]. Acetylation of K6 on ubiquitin itself has been shown to inhibit the assembly of K48-linked ubiquitin [43].

Ubiquitination shows no clear consensus sequence, unlike other PTMs including phosphorylation [46] and N-linked glycosylation [47]. The target specificity for E3 ligases is determined by more complex factors, such as the phosphorylation or glycosylation state of the protein [48]. The target specificity of DUBs is even less clear. However, it is still possible that the abundance of amino acids around a particular lysine will affect the likelihood that ubiquitination occurs at that residue. Accordingly, it has been possible to train machine learning models for the prediction of ubiquitination sites based on amino acid composition around the modified lysine [49]. Whilst a few early learning algorithms used on the order of ∼200 sites [50], this has been increasing in the last decade [49] with the UbiProber model [51] trained on 11,500 sites for humans, mice, and *S. cerevisiae* proteins. The model supports cross-species prediction, in which the training data from one organism is used to predict ubiquitination sites in another. Recently, a deep learning model was trained with over 50,000 ubiquitination sites producing one of the best performances to date, with a prediction accuracy of almost 90% [52]. While ubiquitin site profiling supersedes use of these models for investigating ubiquitination in model organisms, these models may provide a prediction of the ubiquitome for other less studied species.

Ubiquitin site profiling under different conditions has given an unprecedented way to study the function and enzymes of the ubiquitin system. Firstly, it has been possible to dissect the substrates of E3 ubiquitin ligases and DUBs, and secondly, many new ubiquitination events and modified proteins have been identified following changes in environmental conditions and stress. The chemical and genetic ablation of ubiquitin system enzymes followed by ubiquitin site profiling is a key method for inferring specific ubiquitination substrates and has been applied extensively for E3 ligases and DUBs (Table 3). Comparison between conditions with either genetic removal, knockdown, inhibition, overexpression, or reconstitution with a non-catalytically active E3 ligase or DUB can reveal how the activity of the protein affects the global ubiquitome. The differences in the resulting ubiquitomes can then be used to predict substrates and processes affected by that protein. However, it is not possible to directly infer substrates from these types of experiments as the activity of the E3 ligase or DUB may affect other components of the ubiquitin system [53] and ubiquitin site profiling experiments provide no information on ubiquitin topology. It remains difficult to validate putative DUB or E3 targets as bona fide substrates of the particular enzyme due to the low stoichiometry of ubiquitination and the pleiotropic effect of ubiquitination. There are a range of experiments for defining E3- and DUB-substrate pairs and networks [54], but the ubiquitomics approach is still very powerful because it shows the direct effect of alteration of the DUB or E3 ligase activity on the ubiquitome.

The assignment of the substrates of DUBs is becoming increasingly important as these enzymes are considered “druggable”. Specific DUB inhibitors are becoming more common and allow for the chemical alteration of DUB activity in cells. This is interesting in drug/target discovery setting, as the alteration of the ubiquitome is the end goal for DUB inhibitor use in cells. This has been exemplified in vivo by the success with USP7 inhibitors for cancer treatment in mice [23,55]. USP7 inhibition causes degradation of MDM2 due to stabilisation of proteasome-targeting ubiquitin chains, which in turn stabilises the tumour suppressor p53. The mapping of DUB substrates will be important for providing new angles to target proteins such as c-MYC which do not have surface features that are suitable for inhibitor development, which may represent as much as 80% of the proteome [56]. c-MYC exemplifies a classically “undruggable” protein [57], however its protein level is regulated by USP28 activity [58] raising the possibility of USP28 inhibition to target c-MYC levels [59,60]. This highlights DUBs as promising alternative drug targets in cancer and other diseases [61,62].

## 4. Limitations of Ubiquitin Site Profiling

Improvements in MS and technology have been driving forward proteomics, with mass spectrometers having greatly increased sensitivity and acquisition speed. However, measurement of low abundance peptides and modifications is still challenging due to the limitations in dynamic range [77]. Even following a K-GG pulldown, GG-modified peptides are still low abundance and typical data-dependent acquisition (DDA) MS workflows are not optimised for the detection of such peptides. In a DDA mode, a mass spectrometer first scans all ions (MS1), then selects the top-N peaks based on abundance for fragmentation and a rescan (MS2). There have been attempts to alter the DDA workflow for detecting ubiquitination such as selecting the top-N peaks for fragmentation, followed by lowest-N ions in the next cycle, which was found to increase measured GG-sites with use of less starting material [78]. One of the ways to compensate for the low abundance of GG peptides and to increase ubiquitome depth has been to use a large amount of starting material. The seminal UbiSite paper used up to 50 mg of protein starting material per condition in triplicate [21], and the CST PTMScan Ubiquitin Remnant Motif Kit recommends using 10–20 mg of protein input per pulldown. There have been titrations reported with varying amount of starting material [29,78] and new sample preparation methods that require less material [29]. For instance, the latest technology from CST, the PTMScan HS Ubiquitin/SUMO Remnant Motif kit (59322), requires only 1 mg of starting material by implementing SDS lysis and S-Trap columns in the workflow.

To reduce the need for large amounts of sample material and increase the sensitivity of detection, going beyond the DDA approach is a clear avenue for ubiquitomics. Targeted proteomics has been applied for quantitation of known ubiquitination sites following mitophagy where sample material was extremely limited [79]. However, the two above-mentioned recent studies on development of DIA ubiquitomics methods show great promise for the ubiquitomics field. For instance, in Steger et al. 30,000 sites were detected in a single run with only 500 μg of protein input [23]. In the future, DIA mass spectrometry may become sensitive enough that ubiquitin remnant enrichment may not be necessary, and stochiometric information on PTM sites can be obtained. Finally, most ubiquitin site profiling studies have used Collision-Induced Fragmentation for MS2 analysis. However, other fragmentation strategies, such as Electron Transfer Dissociation, can provide alternative coverage of K-GG sites [80,81], which may give deeper or higher confidence ubiquitome data.

Spatial proteomics has begun to globally map protein localisation in cells, but the mapping of PTMs in each compartment has been lagging. The Human Cell Atlas [82] and information from other techniques [83] suggest that up to 50% of proteins are found in multiple cellular compartments. In each compartment exists a unique set of DUBs [6] and E3 ligases, suggesting that a protein located in multiple compartments could exhibit different ubiquitination landscapes in each compartment. This has been shown in principle for a protein located in both the nucleus and cytosol, where ubiquitination in each compartment occurs on different residues and leads to different outcomes [84]. While there have been enrichments of organelles in some ubiquitomic experiments [31,32,70], there have been no comparisons between organelles, which may be an interesting direction of research for the ubiquitomics field.

Almost all ubiquitin site profiling has focused on lysine modification. However, there are more and more examples of non-lysine ubiquitination in a variety of contexts [85] and there are few ubiquitomics methods for mapping these modifications. A few E3 ligases in humans have been discovered to possess esterification activity, ubiquitinating serine and threonine residues [86,87]. Several studies have now mapped N-terminally ubiquitinated proteins [16,21] through a variety of approaches. The most notable of these is the UbiSite method, which can enrich for ubiquitinated material regardless of the modified residue. Treatment with proteasome inhibitor did not cause accumulation of N-terminal ubiquitin modification, suggesting this chain type has a role outside of proteasome degradation [21]. Systematic mapping of serine/threonine ubiquitination sites is yet to be reported.

Despite the large number of identified ubiquitination sites, with over 120,000 sites in the PhosphositePlus database as of May 2020 [24], there are few identified E3- and DUB-substrates pairs or networks. A recent database contained 1806 E3-substrate pairs based on high-throughput databases of protein interactions, and this information was used to train a E3-substrate predictor [88]. However, there are many studies which profile E3- and DUB-substrate pairs and there is a need to aggregate this information in databases to form a more complete picture of the ubiquitin system. There is still extremely limited functional annotation of ubiquitination sites. Ubiquitomics suffers from the same difficulties as phosphoproteomics, where most reported phosphorylation sites either do not have an associated kinase or have unknown function [89]. There are over 235,000 human phosphorylation sites in the PhosphositePlus database as of May 2020, but only 8097 tsites have a reported function. This problem is even more pronounced for ubiquitomics. Among the 100,000 human ubiquitination sites, only 105 have an annotated function in the database. Whilst this as much shows the limitation of the database, it shows the massive task that the field will face in deciding which of these sites have real biological function and deciphering their roles.

Over half of known ubiquitination sites are only reported in 1 out of the 14 studies that have been compiled by the PhosphoSitePlus database (Figure 1C). Many of these sites have been detected in an unphysiological context under conditions such as proteasome and DUB inhibition, resulting in sites which may not be ubiquitinated in a physiological setting. Proteasome inhibition causes ubiquitination levels of proteins to increase, but this does not cause their accumulation, as the inhibition of protein degradation prevents protein synthesis [21]. False positive assignment of ubiquitination sites due to errors in mass spectrometry have long been known, including issues with false discovery rates and spectral assignment making it difficult to localise ubiquitination sites to a particular residue [90]. Artefactual modifications arising from the sample preparation can be mistaken for characteristic +114.08 Da modification of the GG remnant [91]. A combination of these errors and aggregation of ubiquitome data from multiple sources leads to an accumulation of false positives in the databases, which will be difficult to screen out.

## 5. Proteomics with Activity-Based Probe Profiling

**Figure 2:**
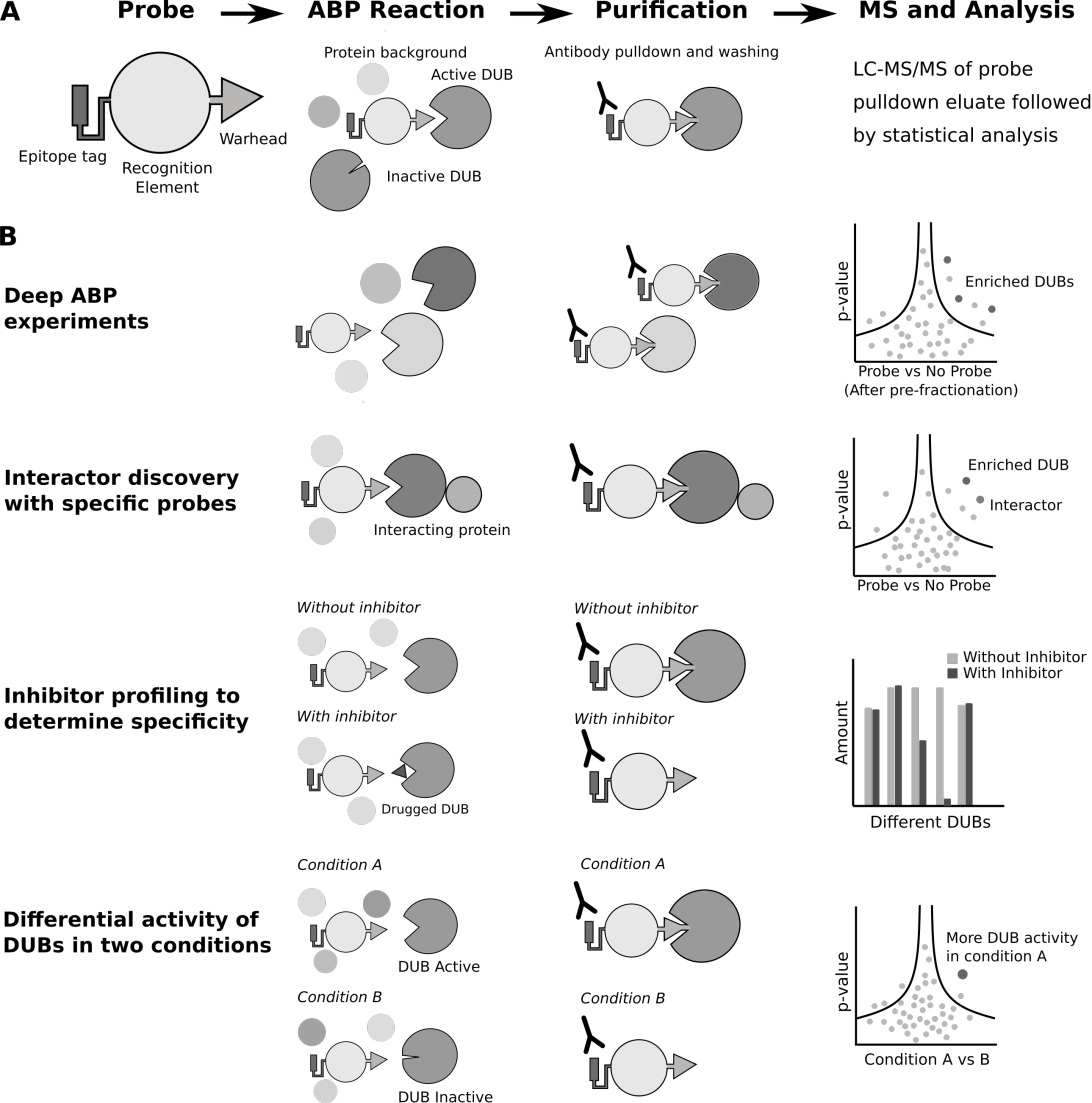
Activity based profiling for DUBs. (**A**) A schematic for an activity-based probe profiling experiment coupled with LC-MS/MS. (**B**) Types of ABPP experiment: (i) catalogue of DUBs to show active DUBs in cells and to discover new DUBs in mammals and other species; (ii) specific probes allow pulldown of DUBs to study their interactomes; (iii) inhibitor profiling can show specificity of DUB inhibitors; and (iv) DUB ABPs can show how DUB activity is upregulated in certain conditions, giving clues to the function of that DUB.

Activity-based probes (ABPs) enable the profiling of active enzymes in a cell, and have been developed for many classes of proteins, especially proteases [92]. A classic ABP contains a specificity domain that targets them to their target enzyme, a chemical warhead that reacts irreversibly with the active site of the enzyme, and an identification moiety that enables the visualization or purification of the probe-enzyme species. Specific ABPs exist for E3 ligases, DUBs, and for the investigation of ubiquitin-like proteins and associated enzymes. The combination of ubiquitin site profiling and ABPs targeting the ubiquitin activitome increases the power of both approaches. ABPs can inform on the relative cellular activity of an E3 ligase or DUB, allowing for specific cellular conditions or inhibitor conditions to be selected and the corresponding effect on the ubiquitome assessed. The first DUB probe was developed in 2001 [93], and the last ∼20 years has seen an explosion in different DUB probe chemistries [94]. Additionally, there are now probes for the HECT [95] and RING [96] domain E3 ligases. DUB ABPs contain three elements: an epitope tag for pulldown and visualization of the probe, a recognition element (typically ubiquitin), and a chemical warhead that reacts irreversibly with the catalytic residue of active DUBs (Figure 2A). These probes have allowed the development of several MS-based methods to further investigate the biology of DUBs (Figure 2B).

The first use of ABPs has simply been to catalogue active DUBs in cells. There are two broad classes of DUBs [6], both of which have specific ABPs. DUBs with catalytic cysteines are most successfully targeted with a Ub-Propargylamine ABP [97], and metalloDUBs, which contain a Zn${}^{2+}$ in their active site, can be targeted by a Ub-probe with a zinc-chelating group as a reactive warhead [98]. The depth of cysteine-targeting DUB ABPs has recently enabled the cataloguing of active cysteine-based DUBs from mammalian tissue culture; proteomic analysis showed that, out of 78 expressed DUBs, 74 reacted with the Ub-Propargylamine ABP [99]. The limited depth of E3 ligase ABPs precludes their use for this type of cataloguing experiments. For example, the E1-E2-E3 ligase cascading probe identified both E1 enzymes, around 20 E2 enzymes and only 2 E3 ligases, out of ∼600 total E3 ligases [100].

ABPs have been used to identify new DUBs in mammals and other species. This has been exemplified by the recent discovery of ZUFSP1 with cysteine-targeting ABPs, a member of a distinct class of DUBs [101,102]. ZUFSP1 was originally thought to be an inactive DUB as it seemed to lack a complete catalytic triad. However, the ABP reacted with the catalytic cysteine which could be identified in MS2 spectra and shown to play a role in DNA repair signalling. ABPs have been used to discover active DUBs in other species, including plasmodium [103] and Leishmania [104].

Ubiquitomes are dynamically regulated by the activity of E3 ligases and DUBs [105], and ubiquitin profiling experiments fail to capture the underlying range of enzymes, the “activitome”, that might be shaping the ubiquitome. Whilst comparison of ubiquitin under different conditions can show which proteins are ubiquitinated during a cellular response, this does not give information on what enzymes are restructuring the ubiquitome. ABPs can be used to investigate which DUBs and E3s change activity during cellular events. For example, UCH-L5 activity was found to increase during Salmonella infection [106], and OTUD3 activity was found to increase during viral infection [107]. In a recent paper with the first RING-targeting E3 ABP, the authors compared normal growth conditions with EGF stimulation, finding increased activity of E3 ligases known to be associated with EGFR signalling [96]. These studies show the potential of ABPs for discovering novel roles of E3 ligases and DUBs in coordinating ubiquitin signalling networks [108].

Variants of the ubiquitin ABPs have allowed a closer inspection of DUB specificity. Di-ubiquitin probes with a non-hydrolysable linkage have been engineered to resemble different ubiquitin chain types [109]. The highly specific probe, Met1-linked diUb probe, exhibits exclusive affinity for OTULIN, known to cleave linear ubiquitin. Using this probe, with and without a pan-DUB inhibitor, it was possible to identify SNX27 as an interactor of OTULIN, enabling the probe to be used to perform a co-IP-like experiment [110]. Finally, DUBs are rising as therapeutic targets. However, the first DUB inhibitors showed poor selectivity [111]. DUB ABPs are now being used extensively to ascertain the selectivity of DUB inhibitors. The development of the specific USP7 inhibitor involved ABP profiling, where cell lysate was incubated with inhibitors followed by ABP profiling [55]. This showed that only the reactivity of USP7 with the probe was affected by the inhibitor. Furthermore, cells can be treated with inhibitors prior to lysis followed by ABP profiling to show target engagement. Currently, this is the main method for studying target engagement of small molecules in a cellular context, allowing the development of much more selective and therefore safer treatments.

## 6. Ubiquitin Chain Topology and the Ubiquitin Code Hypothesis

**Figure 3:**
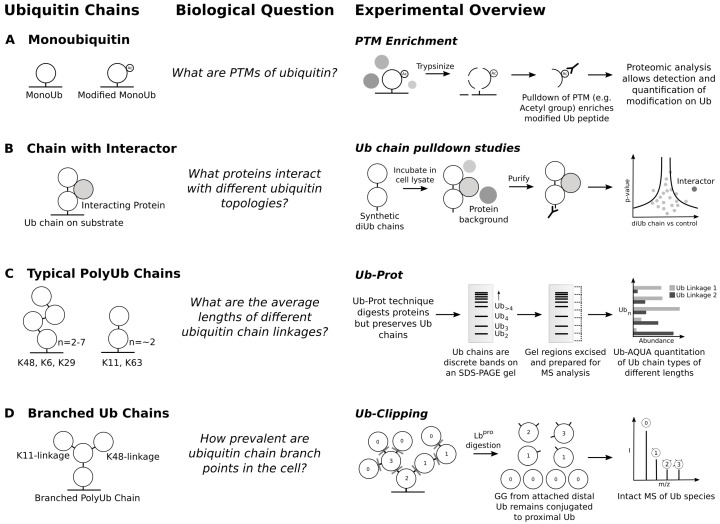
MS advances in deciphering ubiquitin chain topology and the ubiquitin code. (**A**) Ubiquitin itself can be modified by a plethora of PTMs which are amenable to detection by MS-methods. (**B**) Novel interactors of ubiquitin chains have been identified through incubation of synthetic diUb chains with cell lysate. Pulldown of these short chains followed by proteomic analysis in the eluate enables detection of novel interactors for different ubiquitin chain types. (**C**) Typical ubiquitin chain structures have been suggested by Ub-Prot, a technique allowing analysis of the length of different ubiquitin chain types. (**D**) Quantitation of ubiquitin chain branch points has been enabled by Ub-Clipping. Lb${}^{pro}$ cleaves ubiquitin between the 74th and 75th amino acid. For ubiquitin monomers in a chain, this cleavage leaves the characteristic GG remnant from a distal ubiquitin (furthest from substrate) conjugated to a proximal ubiquitin. The number of GG remnants can be detected by intact MS suggesting the degree of branching.

Focusing on ubiquitination sites and activity alone misses out on the complex nature of ubiquitin chain topology. Ubiquitin can be modified extensively, most importantly on its own lysine residues, allowing the formation of polyubiquitin chains. Ubiquitin has seven internal lysine residues and can also be modified with ubiquitin at its N-terminus, allowing for eight basic ubiquitin chain types [4]. However, it has become increasingly clear that there exists a wide possible range of ubiquitin chain structures. A single ubiquitin monomer in the chain can be ubiquitinated multiple times, generating hybrid chains with multiple types of ubiquitin linkage [112]. There are important quantitative questions in the field: What modifications exist on ubiquitin itself? What is the abundance of each ubiquitin chain linkage? What proportion of ubiquitin chains in the cell are branched? What proteins are modified by each chain type? MS tools have been instrumental in the research around these questions (Figure 3).

The huge variety in possible ubiquitin chain topologies and modifications has given rise to the idea of the “ubiquitin code”. This idea was first formalised by Komander and Rape in 2012 [113], suggesting that “ubiquitylation can act as a code to store and transmit information” through the assembly of different ubiquitin chain types and modifications. There are many signals associated with different ubiquitin chain structures [114]. The picture is further complicated by the existence of hybrid ubiquitin/Ubl chains, again hugely increasing the diversity of possible structures [115]. Borrowing from the language of the histone code hypothesis [116], E3 ligases act as “writers”, DUBs as “erasers”, and ubiquitin binding proteins as “readers”.

Beyond showing the existence of the varied ubiquitin chain topology, proteomic tools have contributed to ideas of the ubiquitin code hypothesis. Proteomic MS approaches have been the starting point for discovering new readers of the ubiquitin code (Figure 3B). Studies of each possible linkage of diUb species [117] and longer ubiquitin chains [118] show that chain types have specific interactomes, supporting the existence of distinct ubiquitin binding proteins for each chain type. Structural biology has shown that each ubiquitin chain type has a distinct conformation, again lending to the idea that each chain type has unique binders [4]. A recent review postulated that the variety of ubiquitin-controlled processes and chain types suggests that more types of ubiquitin binding domain should exist [119]. While branched ubiquitin chains are common, there are no known binders of any type of ubiquitin branch point.

Ubiquitin can be modified with many more PTMs, further increasing the complexity of the ubiquitin code. MS analysis of ubiquitin through pulldown studies of various PTMs (Figure 3A) have shown that ubiquitin can undergo extensive modification [120], including phosphorylation, acetylation, SUMOylation, deamidation, and ADP-ribosylation. In human ubiquitin, there are eight phosphorylation sites, the best studied of which is pS65, involved in mitophagy signalling [121]. Acetylation of K6 has been reported to negatively regulate formation of K11, K48 and K63-linked ubiquitin chains [43]. Other modifications reflect inhibition or subversion of the ubiquitin system by infectious agents. The glutamine (G40) of ubiquitin was reported to be deamidated by the deaminidase CHBP (Cif homologue from *Burkholderia pseudomallei*) during Burkholderia infection [122]. This blocks the synthesis and elongation of polyubiquitin chains, which dysregulates the eukaryotic ubiquitination machinery. Finally, *Legionella pneumophila* ADP-ribosylates ubiquitin in order to conjugate ubiquitin to Rab small GTPases, enabling ubiquitination independent of the eukaryotic E1 and E2 enzymes [123].

The ability to purify endogenously expressed ubiquitin chains with Tandem Ubiquitin Binding Entities (TUBEs) was a major step forward in studying ubiquitomes [124]. Since their development in 2009, there has a been a large expansion in types of TUBEs and there are binders for Met1-, K27-, K48- and K63-linked chains [125]. Several proteomic workflows have been used to identify proteins modified with these chain types [126,127]. However, the use of TUBEs is still limited by the pulldown of non-ubiquitinated material and the lack of precise ubiquitination site information. A combination of TUBE pulldown followed by trypsinisation and a K-GG pulldown would allow site specific modification data for different chain types, adding another layer of depth to the ubiquitome. This has been attempted in yeast with a K63 TUBE followed by a K-GG pulldown, identifying almost 1000 sites that are likely modified by K63-linked chains [38]. However, this method required lysate containing over 200 mg of protein, so it will be difficult to scale this up for mammalian tissue culture.

Ubiquitin chain type abundance has been estimated with bottom-up targeted proteomics approaches using heavy labelled synthetic GG-modified ubiquitin peptides as a reference for ubiquitin absolute quantitation (Ub-AQUA) [128], using Parallel Reaction Monitoring (PRM) to ascertain the abundance of different modified ubiquitin tryptic peptides. This showed that all chain types are present in yeast. This method has been applied in human cells, again showing the presence of all chain types [129]. Ub-Prot is a recent method to examine both lengths and linkage types of ubiquitin chains in yeast [130]. By pulling down ubiquitin chains with a TUBE that is resistant to trypsin, followed by trypsinisation, Ub-Prot enables isolation of ubiquitin chains from cells. By separating out the purified ubiquitin chains on an SDS-PAGE gel then preparing different regions of the gel lane for Ub-AQUA PRM, it is possible to estimate the abundance of different chain types at different molecular weights. This in turn can be used to infer the length of typical ubiquitin chains of different linkage type. K48-, K6- and K29-linked chains have between two and seven ubiquitin monomers, compared with K63- and K11-linked chains containing typically no more than two ubiquitin monomers (Figure 3C).

However, Ub-AQUA cannot provide information about the abundance of hybrid ubiquitin chains with multiple linkage types and branch points. There have been several examples of hybrid ubiquitin chains in biological processes, including Met1/K63-linked chains in MyD88-driven inflammation [131], K11/K63-linked being required for MHC I internalisation [132,133], and K11/K48-linked chains enhancing proteasomal degradation [134]. Some E3 ligases generate hybrid chains in vitro [114]. Ubiquitin Chain Restriction (UbiCRest) analysis has shown that hybrid or branched chains can be found on other substrates [135]. However, there is still speculation about the prevalence of hybrid chains in the cell, and their potential involvement in other processes.

MS analysis offers a higher vantage point. While complete trypsinisation of ubiquitin chains destroys their architecture, limited trypsinolysis preserves some of the structure of the chains and enables middle down MS. This has been applied to ubiquitin chains generated in vitro [136,137]. Two separate MS-based methods have estimated that around 10–20% of ubiquitin chains contain a branch point in cells. Purification of ubiquitin chains from cells with TUBEs followed by limited trypsinolysis and middle down MS showed that 1–2% of ubiquitin monomers are branched in ubiquitin chains, increasing to 4% on inhibition of the proteasome [138]. Ub-Clipping is a powerful new technique for probing both modifications of ubiquitin and chain architecture (Figure 3D) [139]. This method involves use of an engineered viral protease from Foot and Mouth Virus, Lb${}^{pro}$, that can cleave ubiquitin at its C-terminus, between R74 and G75, leaving behind a GG remnant on a substrate protein. If a ubiquitin is found to have a GG remnant following Lb${}^{pro}$ treatment by intact-MS, that ubiquitin must itself have been ubiquitinated. Two GG remnants imply the ubiquitin must be a branch point in a ubiquitin chain. By combining TUBEs with Ub-Clipping, the authors found that 4–7% of ubiquitin is modified with two separate GG remnants, suggesting that 10–20% of ubiquitin chains contain a branch point. Ub-Clipping has been used to study the ubiquitin chain architectures and modifications that occur in mitophagy, revealing that only ubiquitin monomers at the end of ubiquitin chains or monoubiquitin modifications are phosphorylated rather than monomers within chains [32].

## 7. Translational Ubiquitomics

As discussed above, the ubiquitin system is involved in most cellular processes. Additionally, it has been reported to be deregulated in certain diseases, most notably in cancer and neurodegeneration. There are many mutations that affect the ubiquitin processing machinery. Mutations and genetic alterations in the K63-specific deubiquitylating enzyme CYLD have been found in patients with cylindromatosis, multiple familial trichoepithelioma, and Brooke–Spiegler syndrome [140]. Inactivating mutations of the deISGylating enzyme USP18 are the cause of type I interferonopathies and auto-inflammation events that lead to the life-threatening pseudo-TORCH syndrome [141]. Mutations in E3 ligases that control cell signalling, DNA repair, and cell cycle progression are common in cancer [142]. For example, loss of activity in the E3 ligase pVHL (an inhibitor of hypoxia signalling) is found in almost all cases of renal carcinoma, due to loss of the allele, mutation, or methylation in its promoter [143]. Beyond cancer, mutations in the E3 ligase Parkin are associated with Parkinson’s disease [144].

Studying how these genetic alterations alter the ubiquitome in patient samples could prove very informative and indeed lead to more effective forms of therapy. Mouse tissue has been used successfully for K-GG profiling studies [20,145]. However, since the required amount of starting material for performing ubiquitin site profiling studies is relatively high and the cellular material coming from patients is normally low, this has been challenging so far. For example, K-GG-based ubiquitin site profiling has been used to describe the ubiquitin landscape in platelets derived from six human donors following stimulation by collagen-related peptide (CRP-XL) [73]. Higher throughput MS methods and a decrease in the amount of sample required will help drive translational ubiquitomics. UbiFast allowed the identification of differentially ubiquitinated proteins in basal and luminal human breast cancer xenografts using as little as 500 μg of protein per condition [29]. The developments in DIA ubiquitomics also allow the use of much less protein input [23]. DUB ABPs have been used on human breast cancer tissue samples and showed increased activity of UCHL1, suggesting this enzyme as a therapeutic target in these cancers [108].

## 8. Discussion

The study of cellular ubiquitomics provides unprecedented details into cell biological processes controlled by ubiquitin and its interplay with other post-translational modifications. The topology of ubiquitin chains clearly dictates the fate of cognate substrates, as formulated by the ubiquitin code hypothesis. However, it is not yet clear to what extent the variability in chain structures reflects distinct versus redundant information.

Novel breakthroughs in ubiquitin biology are interlinked with technical advances, and constant developments in chemical and analytics tools are permitting deeper and faster profiling analysis. This includes higher-throughput proteomics permitting many tens to hundreds of samples to be analysed in single experiments. New MS platforms, developments in liquid chromatography, and new MS approaches such as DIA reflect technical efforts towards such a goal.

We have more ubiquitomics data than ever. The compilation of this data and studies is a starting point, but there is little functional annotation of these sites and there is no comprehensive database of DUB and E3 substrates. To better understand ubiquitination in the disease setting, the generation of a database compiling disease ubiquitomics data, mutations in ubiquitinated sites in disease, and their associated modifiers could be a useful resource for diagnostics and for the generation of newer therapies targeting the ubiquitin system.

Implementing the knowledge gained from ubiquitomics into the clinic represents the ultimate challenge for the field. The study of the ubiquitome in disease could be helpful to identify biomarkers, resistance mechanisms, and potential co-treatment opportunities. Since DUB activity can reflect aberrant signalling, studying the DUB “activitome” in disease could also help to better understand its aetiology and unveil new DUB targets. ABPs targeting DUBs and also conjugating enzymes will not only permit the discovery of DUB/E3 ligase activity and substrates, but also allow the testing of small molecule inhibitors as part of a drug discovery pipeline towards applications in the clinic. ABPs have already led to the development of highly specific DUB inhibitors that have promising anti-tumour effects in vivo.

In summary, ubiquitomics has significantly evolved in the last decade and allowed us to better understand the biological meaning of the ubiquitin modification. However, there are still big challenges for the field. Promising technological developments and dedicated databases will help us to untangle the complexity of ubiquitination and to discover novel ways of targeting the ubiquitination machinery in disease.

## Figures and Tables

**Table 1:** Types of ubiquitomics experiment.

Aim	Sample Requirement	Enrichment Method	MS Factors and Analysis	Example
Deep ubiquitome	Up to 50 mg cell culture	UbiSite	Prefractionation to increase depth	[21]
Multiple PTMs	1–20 mg cell culture	UbiSite or K-GG with additional PTM pulldowns	Multi-Omic data analysis	[31]
Multiple conditions	0.5–20 mg cell culture	K-GG or TUBE	Use of SILAC or TMT— in-solution or on-bead	[19,29]
Chain type specific	1–200 mg cell culture/yeast	TUBE, possible to combine with K-GG	-	[38]
Low abundance modifications	<1 mg lysate	K-GG	Use of DIA to increase MS sensitivity	[22,23]

**Table 2:** List of resources for ubiquitination site identification.

Databases	Information	Reference
PhosphositePlus Database	Most comprehensive database for protein ubiquitination including most recent studies	[24]
Protein Lysine Modification Database (PLMD)	Contains information on lysine ubiquitination and on other lysine modifications. Potential for investigating PTM crosstalk	[41]
Mammalian Ubiquitination Site Database (mUbiSiDa)	A database of ubiquitination sites assembled in 2013	[44]
Ubiquitin and Ubiquitin-like conjugation Database (UUCD)	A database of actual and predicted ubiquitin and Ubl associated machinery in several species	[45]

**Table 3:** Examples of ubiquitin site profiling studies to investigate substrates of the ubiquitin machinery and for the discovery of ubiquitinated proteins following specific stimuli.

Ubiquitin Modifying Enzyme	Study Details	Reference
Cullin Ring Ligases	K-GG, CRL inhibition	[18]
SPOP	K-GG, SILAC, mutant and overexpression	[33]
Parkin	K-GG, inactive mutant	[28]
	K-GG, inactive mutant	[32]
LZTR1	K-GG, knockout	[63]
HUWE1	K-GG, knockdown	[64]
Skp2	TUBE, overexpression	[65]
USP7	K-GG, DIA-MS, inhibitor	[23]
USP9X	K-GG, knockdown	[66]
USP22	K-GG, knockdown and overexpression	[67]
USP30	K-GG, knockdown	[68]
	K-GG, knockout	[32]
	K-GG, inhibitor	[69]
	K-GG, knockout and inhibitor	[70]
USP32	TUBE, knockdown	[71]
*Stimulus*		
UV-induced DNA damage	K-GG, SILAC	[27]
UV- and radiation-induced DNA damage	K-GG, SILAC	[31]
TNF signalling	K-GG, SILAC	[72]
Cell cycle synchronisation	K-GG, DIA-MS	[23]
Lenalidomide treatment	K-GG, UbiFast/TMT	[29]
CRP-XL signalling	K-GG	[73]
Photosensitiser treatment	K-GG	[74]
Proteasome inhibition	K-GG, SILAC	[18]
	K-GG, SILAC	[75]
	UbiSite	[21]
Muscle atrophy	K-GG, time course examining mouse muscle ubiquitome following atrophy	[76]
